# A web-based augmented reality system for orthodontic biomechanics: perceived usability and acceptability among dental students

**DOI:** 10.1186/s12903-026-08004-3

**Published:** 2026-03-07

**Authors:** Xiao Li, Tia Farisha Shaharul Miza, Syaidatul Salmah Nurbalqis Saiful, Gururajaprasad Kaggal Lakshmana Rao, Fakhitah Ridzuan, Hasnah Hashim, Zuhaila Ismail, Nordin Zakaria, Norehan Mokhtar

**Affiliations:** 1 Department of Dental Science, Advanced Medical and Dental Institute, Universiti Sains Malaysia, Bertam, Kepala Batas 13200 Malaysia; 2 Department of Computing, Universiti Teknologi PETRONAS, Bandar Seri Iskandar, 32610 Malaysia; 3 Department of Orthodontics, Penang International Dental College, NB Tower 5050, Jalan Bagan Luar, Butterworth, 12000 Malaysia; 4 Faculty of Data Science and Computing, Universiti Malaysia Kelantan, City Campus, Pengkalan Chepa, Kota Bharu, Kelantan 16100 Malaysia; 5 Department of Dental Public Health, Faculty of Dentistry, Asian Institute of Medicine, Science and Technology (AIMST) University, Bedong, Kedah 08100 Malaysia; 6 Department of Mathematical Sciences, Faculty of Science, Universiti Teknologi Malaysia (UTM), Johor Bahru, Johor 81310 Malaysia; 7 Dental Simulation and Virtual Learning Research Excellence Consortium, Department of Dental Science, Advanced Medical and Dental Institute, Universiti Sains Malaysia, Bertam, Kepala Batas 13200 Malaysia

**Keywords:** Orthodontic education, Educational technology, Augmented reality, System usability scale, Technology acceptance model

## Abstract

**Introduction:**

The orthodontic biomechanics of tooth movement form the foundation of orthodontics and is essential for successful treatment outcomes. However, the traditional teaching method faces challenges, such as the complexity of concepts and difficulties in explaining three-dimensional (3D) tooth movement. To address these challenges, a web-based augmented reality (AR) system for orthodontic biomechanics (WAR Orthobiomechanics) was developed as a blended learning tool.

**Aims:**

To evaluate the user satisfaction and behavioural intention of the WAR Orthobiomechanics system.

**Materials and methods:**

A total of 56 undergraduate dental students from Years 3 to 5 at three institutions in Malaysia participated in this study. The System Usability Scale (SUS) and Technology Acceptance Model (TAM) were employed to assess participants’ perceived usability and acceptability. Data were collected between July and August 2023 and analysed using descriptive statistics, independent *t*-test, one-way ANOVA analysis, and Pearson correlation analyses.

**Results:**

Both SUS and TAM indicated positive user experiences. The overall SUS score was 68.97 ± 12.87 (moderate to good). The TAM showed satisfactory acceptability with a mean score of 76.92 ± 13.22. Pearson correlation analyses showed a moderate positive association between SUS and the overall TAM score (*r* = 0.493, *p* < 0.01), indicating concurrent validity between perceived usability and technology acceptance.

**Conclusion:**

The web-based AR system demonstrated acceptable usability and positive student acceptability in supporting the learning of orthodontic biomechanics. The evaluation results indicate that the system is feasible as a supplementary instructional tool.

## Introduction

Orthodontic treatment involves applying orthodontic force on the dental crowns to realign misaligned teeth. A thorough understanding of the biomechanics of orthodontic tooth movement (OTM) is essential for effective and safe orthodontic treatment [8, 34]. Misapplication of biomechanical principles may lead to undesirable movement or damage to periodontal tissue [25]. A solid grasp of orthodontic biomechanics may reduce treatment time by enabling more efficient tooth movement [50]. Therefore, the accurate application of biomechanical principles is crucial to achieving successful orthodontic outcomes.

Based on the Curriculum Guidelines for Orthodontics issued by the American Dental Education Association (ADEA) [11], the biomechanics of OTM and types of tooth movement should be an important element of undergraduate dental education. A modified Delphi study by Ferrer et al. [16] further emphasised the need for novice orthodontists to *understand* key subtopics within OTM, as outlined by the revised Bloom’s taxonomy [4]. Furthermore, the World Federation of Orthodontics (WFO) guidelines highlight the importance of establishing strong foundational biomechanical competence prior to advanced clinical training [2]. Collectively, these guidelines underscore the need for a solid foundation in orthodontic biomechanics during undergraduate education.

However, learning the biomechanics of OTM presents several challenges for dental students and practitioners [17, 18]. The subject matter is inherently complex, requiring the application of mechanical principles to biological systems. Students often struggle with foundational concepts such as force, torque, stress, and strain, which are crucial before advancing to more complex topics. Moreover, the biomechanics of OTM integrates knowledge from various disciplines, including physics, biology, anatomy, and materials science, making it challenging to understand fully [2]. In addition, OTM involves complex 3D structural changes, which demand strong spatial reasoning abilities; however, conventional teaching methods offer limited capacity to visualise these 3D dynamics effectively [48]. Consistent with these limitations, an e-Delphi study conducted by Kaggal Lakshmana Rao et al. [27] revealed that current teaching methods lack effective visualisation, leading to difficulty in understanding orthodontic biomechanics and dental structure responses. Similarly, Poblete et al.’s study [42] revealed that 70% of students and instructors viewed OTM biomechanics as an area where 3D digital resources would be particularly valuable.

Given these challenges and the recognised need for improved visualisation, improving instructional strategies for teaching orthodontic biomechanics has become increasingly important. Numerous studies have explored the use of AR or virtual reality (VR) to provide interactive learning experiences and enhance clinical preparation [10, 24, 26, 28]. Although simulation-based teaching shows promising educational benefits in orthodontics [6, 39], few studies have applied these technologies specifically to orthodontic biomechanics, indicating a gap in current educational resources [44].

Therefore, this study introduces the WAR Orthobiomechanics system, an AR-enhanced learning tool designed to address the visualisation challenges inherent in teaching orthodontic biomechanics. AR enables the presentation of complex 3D movements and interactive force systems in an intuitive and immersive manner, offering dental students opportunities to manipulate tooth movement scenarios [14]. By allowing learners to actively engage with virtual models, AR provides flexible, remote access to learning resources.

To evaluate students’ initial experiences with this newly developed system, the study adopts the reaction level (Level 1) of the Kirkpatrick Model. As the foundational tier of the framework, the reaction level focuses on learners’ immediate perceptions, satisfaction, and acceptance, which are critical indicators of whether an instructional innovation is usable, engaging, and aligned with educational needs [30]. Assessing student reactions is especially appropriate for a preliminary study, as early perceptions often influence willingness to continue using the system and inform subsequent refinement [30].

In line with the focus on students’ initial reactions, this study assesses the usability and acceptability of the WAR Orthobiomechanics system. Usability is a core component of learner experience and influences the extent to which students can interact effectively with digital educational tools [29, 38]. Acceptability further reflects students’ perceptions of usefulness and their willingness to engage with the system [45], both of which are essential when introducing a new instructional technology. To capture these complementary aspects, the study employs two widely established measures: the System Usability Scale (SUS) for perceived usability and the Technology Acceptance Model (TAM) for perceived usefulness (PU) and perceived ease of use (PEOU). Using both instruments provides a comprehensive understanding of how students experience the system during its initial implementation.

## Materials and methods

All participants were required to sign a consent form, confirming their understanding of the research objectives and tasks. Ethical approval was granted by the Human Research Ethics Committee (HREC) of Universiti Sains Malaysia (USM/JEPeM/21110756).

### Content design of WAR Orthobiomechanics

**Fig. 1:**
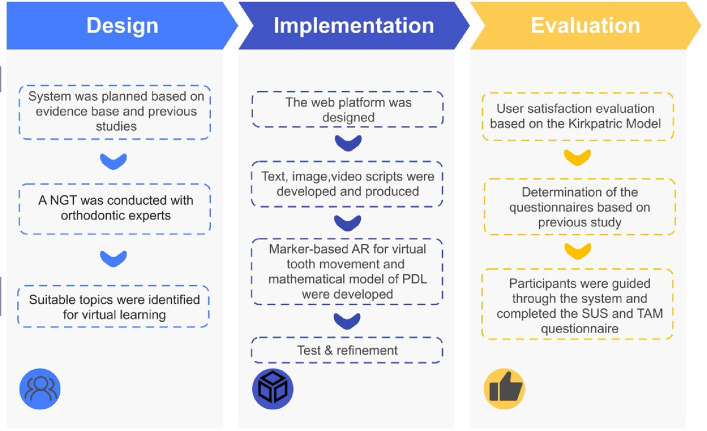
WAR Orthobiomechanics development process. *AR: Augmented reality; PDL: periodontal ligament; NGT: Nominal Group Technique; SUS: System Usability Scale; TAM: Technology Acceptance Model

The content of the WAR Orthobiomechanics system was designed using the Nominal Group Technique (NGT), which identified key topics considered most beneficial for virtual learning in orthodontic biomechanics [44]. Orthodontic experts prioritised physiology of tooth movement, tooth movement (definition, types, theory, and indications), and force systems [44]. Accordingly, the system content was organised into five sections: types of tooth movement, tissue responses to orthodontics, orthodontic wires, anchorage, and simulated tooth movement. Figure 1 outlines the content development workflow.

### Development of the website

#### Responsive web platform

**Fig. 2:**
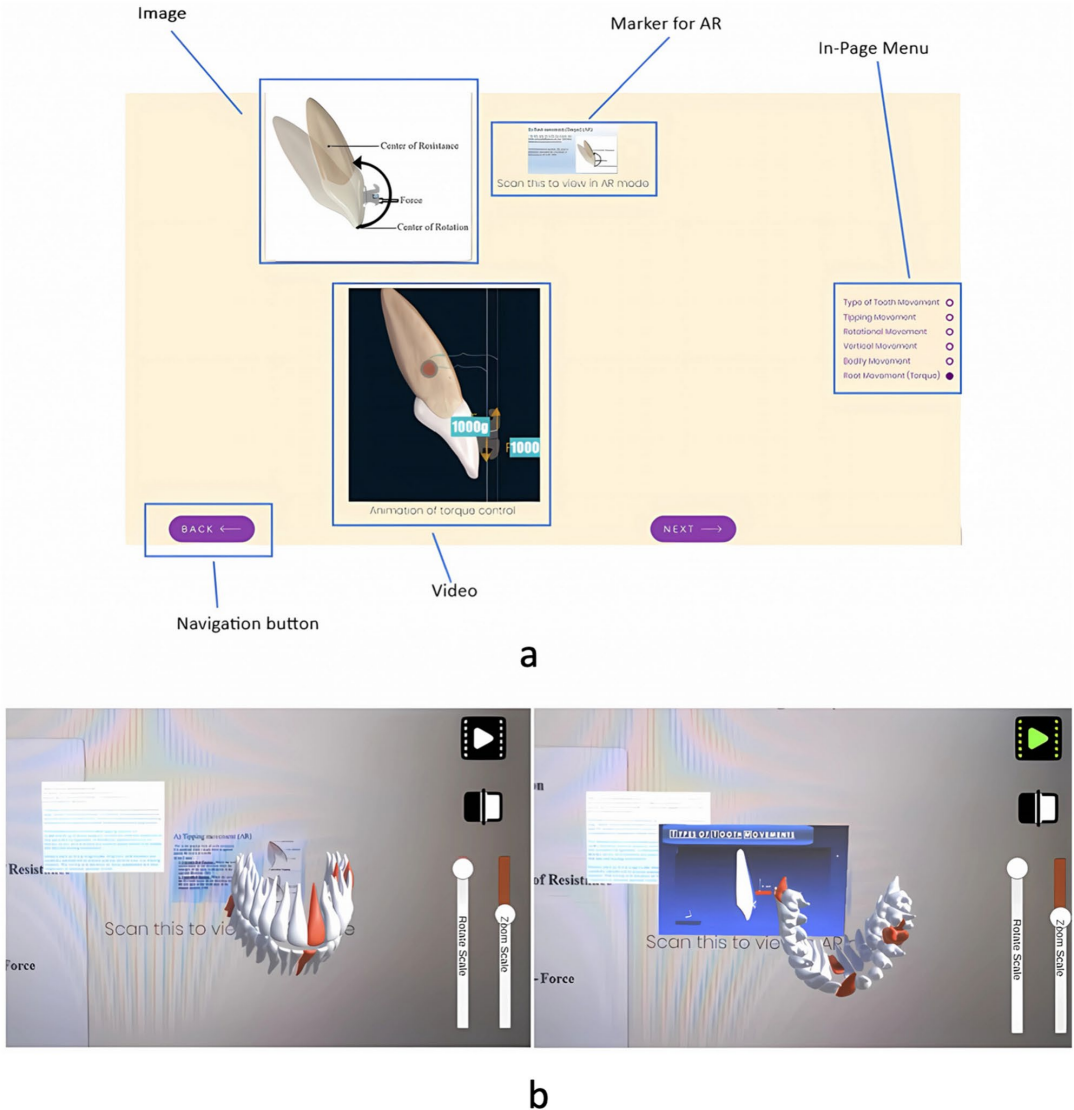
**a** Elements in the website **b** Snapshots of the marker-based AR system

- The platform uses responsive web design (RWD) to ensure consistent access across laptops, tablets and smartphones. The layout automatically adjusted to screen size to optimise usability and visual clarity.
- The system integrates text, animations and AR modules into a single learning interface (Fig. 2).

#### Marker-based AR module

- Data processing and 3D model preparation
  A cone-beam computer tomography (CBCT) dataset was segmented using 3D Slicer, and tooth models were exported in STL format for use in Unity.
- AR tracking and visualisation
  Vuforia was used to generate an image-target marker and enable six-degree-of-freedom tracking for real-time visualisation of the dentition model.
- Tooth movement simulation
  A physics-based engine written in C# enabled the simulation of tipping, bodily movement, and torque (Fig. 2).
  Simulations were checked against clinical records and laboratory measurements to ensure plausibility.
- Mobile implementation
  The Android version was optimised using Unity’s IL2CPP backend to improve execution speed.
  Interface scaling and touch interactions were adjusted to support various screen resolutions.
  The final application was compressed and obfuscated to ensure stable performance on a wide range of devices.

#### Periodontal ligament stress analysis module

- The periodontal ligament (PDL) stress analysis module was developed using finite element analysis (FEA) to illustrate stress and strain responses under orthodontic loading.
- COMSOL Multiphysics was used to model the PDL as a deformable layer between the root and alveolar bone.
- Orthodontic force was applied digitally to simulate stress distribution and displacement patterns.
- Output visualisations included von Mises stress maps and displacement fields.

### Perceived usability and acceptability evaluation of WAR Orthobiomechanics

#### Study design

This study employed a cross-sectional design, involving students from three dental schools in Malaysia: Universiti Sains Malaysia (USM), Penang International Dental College (PIDC), and Universiti Sains Islam Malaysia (USIM).

The inclusion criteria were as follows: 1) Undergraduate dental students in Years 3 to 5 who had attended the orthodontic biomechanics lecture at their respective institutions, and 2) Students who have access to the WAR Orthobiomechanics system using a compatible device. Students who did not complete the consent form were excluded from the study. A purposive sampling method was used.

#### Perceived usability evaluation

The SUS was employed to assess the perceived usability of the WAR Orthobiomechanics system. SUS is a widely adopted and validated instrument for evaluating digital systems across diverse contexts [7] and remains reliable even with small sample sizes [22, 32, 49, 51].

The SUS consists of 10 statements presented in an alternating positive and negative format. Participants rate each item on a five-point scale, ranging from “Strongly Disagree” to “Strongly Agree” [51]. In this study, the questionnaire included five demographic questions and 10 SUS items, with “WAR Orthobiomechanics” substituted for the generic term “system.” Scores were calculated following the standard SUS scoring procedure, producing a total score ranging from 0 to 100. Usability levels were interpreted using the curved grading scale (CGS) [33] (Table 1) and the adjective rating scheme proposed by Bangor et al. [5].

**Table 1:** The curved grading scale for SUS interpretation [33]

Range of SUS Score	Grading	Percentile Range
84.1–100	A +	96–100
80.8–84.0	A	90–95
78.9–80.7	A −	85–89
77.2–78.8	B +	80–84
74.1–77.1	B	70–79
72.6–74.0	B −	65–69
71.1–72.5	C +	60–64
65.0–71.0	C	41–59
62.7–64.9	C −	35–40
51.7–62.6	D	15–34
0.0–51.6	F	0–14

#### Acceptability assessment

Acceptability of the WAR Orthobiomechanics system was evaluated using the TAM, which explains technology adoption through PU, PEOU, attitude toward use (AT), and behavioural intention (BI) [12]. TAM has become a widely accepted model for evaluating the acceptability of information systems due to its versatility, simplicity, and robustness [1]. Research further supports TAM and its variations as a significant scientific framework and a reliable model for assessing the adoption of new technologies in educational settings [21].

In this study, a TAM questionnaire developed by Ghani et al. [19] was selected because it is validated for mobile learning contexts. The questionnaire consisted of five demographic items and 20 TAM items across four dimensions (PU: 5 items, PEOU: 6 items, AT: 5 items, BI: 4 items), each rated on a 7-point Likert scale. An open-ended question captured additional user feedback. The final TAM score (0–100) was computed using the established scoring procedure described by Pal and Vanijja [41], with higher scores indicating greater acceptability.

#### Data collection

To ensure a meaningful learning experience, participants were instructed to download the AR application and view a demonstration video before use. Each student had 90 min to engage with the system, after which SUS and TAM questionnaires were administered via Google Forms. Each questionnaire should be completed within 15 min. Participation was voluntary and confidential.

#### Statistical analyses

Cronbach’s Alpha coefficient was calculated to ensure internal consistency for both SUS and TAM items. Descriptive statistics, including mean, standard deviation (SD), and percentages, were computed to summarise participant characteristics and overall usability and acceptability scores.

Independent *t*-tests were conducted to explore differences by gender, institution, and educational level. USM was analysed separately due to its direct involvement in system development, which may have influenced students’ exposure and familiarity. In contrast, USIM and PIDC were grouped as external institutions with similar evaluation conditions. Students were categorised into preclinical (Year 3) and clinical stages (Years 4 & 5) based on their educational level. One-way ANOVA was used to investigate differences among various device types. To assess the concurrent validity between the SUS and TAM, as well as the internal associations within the TAM framework, Pearson’s correlation analyses were performed.

All analyses were conducted using IBM SPSS Statistics version 29.0, and statistical significance was set at *p* < 0.05.

## Result

### Participant demographics

Out of 72 eligible students, 56 participated in this study (response rate: 77.8%). All participants in this study successfully followed instructions, engaged with the required learning content, and completed both the SUS and TAM questionnaires. Table 2 provides a comprehensive overview of the respondents’ demographics. Among the participants, 34 (60.7%) were female, and 22 (39.3%) were male, with an even split between preclinical (Year 3) and clinical (Years 4 and 5) students. The participant group included 25 students (44.6%) from USM and 31 (55.4%) from other institutions. During the usability test, 27 participants (48.2%) used a laptop, 15 (26.8%) used a mobile phone, and 14 (25.0%) used an iPad or tablet.

**Table 2:** Descriptive statistics of the respondents’ characteristics

Variable	Frequency (*n* = 56)	Percentage (%)
Gender		
Male	22	39.3
Female	34	60.7
Institution		
USM	25	44.6
Others (USIM & PIDC)	31	55.4
Educational level		
Preclinical phase	28	50.0
Clinical phase	28	50.0
Types of devices use for marker-based AR system		
Laptop	27	48.2
Mobile phone	15	26.8
Ipad/Tablet	14	25.0

### Reliability and validity of SUS and TAM

The SUS demonstrated acceptable internal consistency (Cronbach’s α = 0.715), exceeding Nunnally’s [40] recommended threshold of 0.70. Reliability was high across all TAM constructs (Table 3).

**Table 3:** Mean ± SD and internal consistency of TAM

Variables	Item	Mean	SD	Cronbach’s Alpha
PU	PU1	5.83	0.90	0.949
	PU2	5.61	0.98	
	PU3	5.78	1.05	
	PU4	6.09	0.78	
	PU5	5.96	0.97	
PU composite mean		5.85	0.86	
PEOU	PEOU1	5.37	1.20	0.926
	PEOU2	5.57	1.05	
	PEOU3	5.52	1.05	
	PEOU4	5.35	1.23	
	PEOU5	5.57	1.11	
	PEOU6	5.76	0.95	
PEOU composite mean		5.52	0.94	
AT	AT1	6.09	0.84	0.913
	AT2	5.87	0.91	
	AT3	5.85	1.01	
	AT4	5.57	1.09	
	AT5	5.87	0.89	
AT composite mean		5.85	0.82	
BI	BI1	5.63	1.06	0.862
	BI2	4.91	1.17	
	BI3	5.15	1.15	
	BI4	4.96	1.21	
BI composite mean		5.16	0.97	

*PU* perceived usefulness, *PEOU* perceived ease of use, *AT* attitude toward use, *BI* behavioural intention

Pearson correlation analyses (Table 4) showed a moderate positive correlation between SUS and the TAM score (*r* = 0.493, *p* < 0.01), indicating concurrent validity between perceived usability and technology acceptance. SUS also demonstrated significant positive correlations with all TAM constructs, including PU (*r* = 0.486, *p* < 0.01), PEOU (*r* = 0.456, *p* < 0.01), AT (*r* = 0.444, *p* < 0.01), and BI (*r* = 0.346, *p* < 0.05). TAM constructs were strongly intercorrelated (*r* = 0.833–0.914, *p* < 0.01), consistent with the theoretical expectations of the TAM model.

**Table 4:** Pearson’s correlation matrix for all measurements

	SUS	TAM	PU	PEOU	AT	BI
SUS	1					
TAM	0.493**	1				
PU	0.486**	0.901**	1			
PEOU	0.456**	0.891**	0.720**	1		
AT	0.444**	0.914**	0.734**	0.697**	1	
BI	0.346*	0.833**	0.624**	0.658**	0.720**	1

*p < 0.05; **p < 0.01 (2-tailed)

### SUS and TAM score

The mean SUS score was 68.97 ± 12.87 (Table 5), corresponding to a grade of C on the CGS [33]. This score suggests moderate to good usability [5].

**Table 5:** SUS and TAM score according to CGS

	*n*	Minimum	Range	Maximum	Range	Mean	SD	Range
SUS	56	47.50	F	95.00	A +	68.97	12.87	C
TAM	56	48.34	-	100.00	-	76.92	13.22	-

The TAM score was 76.92 ± 13.22 (Table 5), indicating that the tool is useful and easy to use for mobile learning platforms [3]. The mean, SD and internal consistency of TAM were presented in Table 3. PU items produced a composite mean of 5.85. The item “Using WAR Orthobiomechanics will enhance the effectiveness of learning” received the highest score of 6.09, suggesting that the system may support students in improving their understanding of biomechanics. PEOU showed a composite mean of 5.52. AT scores averaged 5.85, with “Studying using WAR Orthobiomechanics is a good idea” rated highest (6.09). BI items had a composite mean of 5.16, with the items “I intend to use WAR Orthobiomechanics heavily” and “I intend to repetitively use WAR Orthobiomechanics as often as possible” receiving slightly lower scores of 4.91 and 4.96, respectively.

SUS and TAM scores were generally comparable across gender, institution, educational level, and device type, with no statistically significant differences observed for overall TAM scores. A significant difference was detected only in SUS scores between preclinical and clinical students (*p* < 0.05) (Table 6).

**Table 6:** SUS and TAM score by demographic characteristics of participants

**Variables**		**SUS** **Mean ± SD**	**TAM** **Mean ± SD**	**PU** **Mean ± SD**	**PEOU** **Mean ± SD**	**AT** **Mean ± SD**	**BI** **Mean ± SD**
Gender	Male (*n* = 22)	68.47 ± 14.02	75.48 ± 14.77	5.78 ± 0.96	5.52 ± 0.94	5.68 ± 0.94	5.04 ± 1.07
	Female (*n* = 34)	69.29 ± 12.34	77.84 ± 12.32	5.90 ± 0.80	5.52 ± 0.96	5.96 ± 0.73	5.24 ± 0.91
	*t*	0.296	1.102	2.100	0.013	1.563	3.189
	*p*	0.589	0.300	0.154	0.909	0.218	0.081
Institution	USM (*n* = 25)	71.25 ± 12.18	76.56 ± 14.58	5.83 ± 0.86	5.46 ± 1.08	5.82 ± 0.89	5.21 ± 1.02
	Others (*n* = 31)	67.21 ± 13.35	77.19 ± 12.37	5.87 ± 0.7	5.57 ± 0.84	5.87 ± 0.77	5.13 ± 0.94
	*t*	0.954	0.306	0.005	1.023	0.152	0.012
	*p*	0.335	0.583	0.946	0.317	0.699	0.915
Educational level	Preclinical phase (*n* = 28)	71.25 ± 10.33	78.07 ± 12.19	5.79 ± 0.73	5.56 ± 0.96	5.93 ± 0.78	5.43 ± 0.87
	Clinical phase (*n* = 28)	67.50 ± 14.26	76.18 ± 14.01	5.89 ± 0.94	5.50 ± 0.95	5.79 ± 0.85	4.99 ± 1.00
	*t*	0.963	0.470	−0.398	0.193	0.564	1.525
	*p*	0.022*	0.460	0.327	0.986	0.361	0.583
Types of devices	Laptop (*n* = 27)	68.33 ± 13.92	75.40 ± 14.46	5.80 ± 0.92	5.42 ± 1.03	5.73 ± 0.94	5.08 ± 0.96
	Mobile phone (*n* = 15)	67.92 ± 11.77	77.52 ± 12.40	5.83 ± 0.72	5.58 ± 0.54	5.97 ± 0.53	5.13 ± 1.18
	iPad/Tablet (*n* = 14)	73.21 ± 11.15	79.85 ± 11.64	6.00 ± 0.91	5.70 ± 0.87	6.00 ± 0.83	5.40 ± 0.72
	*F*	0.442	0.406	0.189	0.344	0.558	0.380
	*p*	0.645	0.669	0.828	0.711	0.577	0.686

*p < 0.05

## Discussion

This study evaluated dental students’ perceptions of the WAR Orthobiomechanics system using established measures of usability and acceptability. Overall, the findings suggest that the system demonstrated moderate-to-good usability (SUS = 68.97) and high levels of acceptability (TAM = 76.92), suggesting its potential as a supplementary learning tool for orthodontic biomechanics.

### Key findings in context

The SUS score (68.97 ± 12.87) indicates that the system’s usability has reached an acceptable level, which falls within the range (63.38—90.33) observed in comparable studies for dental AR applications [28, 31, 47, 52]. However, the wide range of SD indicates that some students encounter difficulties in application, as shown in Table 5, where the lowest SUS score reached 47.50. Prior research has shown that SUS scores for educational AR tools may be lower during early adoption due to novelty effects, limited prior exposure, and increased cognitive load during complex visualisation tasks [13, 15, 36].

The TAM results support students’ perceived acceptability, with high scores for PU (5.85) and positive attitudes toward the 3D simulation system (5.85). Previous studies have demonstrated that students show stronger engagement with 3D models compared to traditional teaching methods [23, 35, 37]. The PU and AT items may indicate that students perceived the system as helpful for visualising 3D tooth movement, a key component of orthodontic biomechanics. PEOU was also rated positively (mean = 5.52), indicating that most students were able to navigate the system and manipulate AR content without major difficulties. PEOU is consistently rated positively by students in digital and AR learning environments, indicating that users generally find these systems navigable and manageable [20, 46]. The slightly lower BI score (mean = 5.16) remains positive overall and may reflect practical constraints such as device compatibility or the need for multiple devices during use.

The moderate positive correlation between SUS and the overall TAM score supports the concurrent validity of perceived usability and technology acceptance (Table 4). Significant correlations between SUS and each TAM construct indicate that students who perceived higher usability also tended to report more favourable acceptance, consistent with previous research on AR and e-learning systems [9, 41, 43].

### Practical implications

In addition to the overall positive usability and acceptability ratings, several subgroup patterns provide practical insights into how the WAR Orthobiomechanics system can be best integrated into orthodontic curricula. Preclinical students scored higher than clinical students on both SUS (71.25 vs. 67.50) and TAM (78.07 vs. 76.18) scores, with a large effect size observed for usability (d = 0.89) (Table 6). This pattern aligns with cognitive load theory, which posits that novices benefit more from structured visualisation tools than advanced learners [10]. In contrast, clinical students with greater prior knowledge may need more clinically contextualised scenarios to maintain their engagement.

Additionally, none of the demographic variables, including gender, institution, educational level, or device type, had a significant effect on SUS and TAM scores, underscoring the system’s broad usability and acceptability across learner groups.

Therefore, WAR Orthobiomechanics may be particularly valuable as a supplementary tool in preclinical teaching, where its 3D visualisation functions can help bridge theoretical biomechanics and early clinical understanding. For instructors, structured faculty development workshops may enhance effective integration, as educator familiarity with these tools is known to improve student outcomes [39]. For more advanced learners, incorporating clinical case simulations may further strengthen the system’s educational relevance.

### System limitations and future improvements

Several limitations were identified based on participant feedback, which could be addressed in future iterations of the system (Table 7). First, iPhone users faced limited access to the 3D module as the AR application is Android-based. Some students found it challenging to use the application, as it requires at least two devices for full functionality, which could be a deterrent. Additionally, the AR system’s performance was occasionally hindered by technical issues such as lagging, and difficulties were reported with the zoom scale functionality, making it less efficient to zoom in and out. To maximise its educational impact, technical optimisations such as improving cross-platform compatibility (especially for iOS), reducing lag, and implementing intuitive interactions like pinch-to-zoom are essential for enhancing accessibility and usability.

**Table 7:** User responses to WAR Orthobiomechanics

Positive feedback	Areas need improvement
This application is indeed very interesting as it helps to enhance my understanding on the tooth movement via the provided 3D module.	Somehow, I find it a bit difficult to use it on-the-go as we need at least two devices to benefit this web-based application.
Very good alternative for learning, especially for things that is hard to just imagine or try to understand it with words only.	For me, AR require better smartphone to view and use the AR techs smoothly, otherwise it can become lagging and stuck sometimes. The idea of web-based application is good but using AR is kind of awkward because the experience could be the deal breaker for me.
It is really a commendable effort and would really be useful for dental student.	I think it’ll be better to zoom in and out and rotating the image by pinching our fingers rather than using the scaling.
Quite easy and interesting to use future!	The zoom scale feature for the app is not working which causes model scale to be small
	I hope the AR also can be IOS-friendly.

To further validate and enhance the WAR Orthobiomechanics system, longitudinal studies should examine whether sustained usage improves academic performance or clinical skill development over time based on the Kirkpatrick model (Level 2).

## Limitations and future work

This study has several limitations. The primary limitation is the small sample size, which restricts the generalizability of the findings to a broader population. Future research with a larger sample size will be conducted to confirm these findings. Additionally, the research model did not account for external factors such as students’ self-efficacy, students’ capacity and experience with AR, system accessibility, and quality. Future studies should incorporate a structural equation model (SEM) to explore the impact of these external factors on PU, PEOU, AT, and BI. Furthermore, this study primarily focused on evaluating the perceived usability and acceptability of the system. However, it did not include objective measurements to assess the system’s impact on actual learning outcomes. Future research should include a comprehensive validation process to evaluate the effectiveness of the system in enhancing learning when compared to traditional learning methods.

## Conclusion

The proposed WAR Orthobiomechanics system demonstrated acceptable usability and positive levels of acceptability, as indicated by SUS and TAM scores. These findings indicate that the system is feasible as a supplementary learning tool in dental education. Student feedback will guide further refinement of the interface, with emphasis on improving system performance and enhancing cross-platform stability. Future studies should address the current limitations and investigate the system’s long-term educational impact to better determine its role in orthodontic teaching.

## Data Availability

The data used and/or analysed during the current study are available from the corresponding author on reasonable request.

## Abbreviations

3D: Three-dimensional
ADEA: American Dental Education Association
AR: Augmented Reality
AT: Attitude Toward Use
BI: Behavioural Intention
CBCT: Cone-Beam Computed Tomography
CGS: Curve Grading Scale
FEA: Finite Element Analysis
HREC: Human Research Ethics Committee
NGT: Nominal Group Technique
OTM: Orthodontic Tooth Movement
PDL: Periodontal ligament
PEOU: Perceived Ease of Use
PIDC: Penang International Dental College
PU: Perceived Usefulness
RWD: Responsive Web Design
SD: Standard Deviation
SEM: Structural Equation Model
SUS: System Usability Scale
TAM: Technology Acceptance Model
USIM: Universiti Sains Islam Malaysia
USM: Universiti Sains Malaysia
VR: Virtual Reality
WAR Orthobiomechanics: Web-based Augmented Reality System for Orthodontic biomechanics
WFO: World Federation of Orthodontics
